# Urban-Rural Differences in Atherogenic Dyslipidaemia (URDAD Study): A Retrospective Report on Diabetic and Non-diabetic Subjects of Northern India

**Published:** 2014-09

**Authors:** Poonam Agrawal, Varikasuvu Seshadri Reddy, Himanshu Madaan, Surajeet Kumar Patra, Renu Garg

**Affiliations:** ^1^Department of Biochemistry, BPS Government Medical College for Women, Khanpur Kalan, Sonepat, Haryana, India; ^2^Department of Biochemistry, Lady Hardinge Medical College, Delhi, India

**Keywords:** Atherogenic dyslipidaemia, Atherogenic index, Diabetes, Urbanization, India

## Abstract

Diabetes and urbanization are major contributors to increased risk factors of cardiovascular diseases. Studying whether atherogenic dyslipidaemia increases with urbanization in type 2 diabetes mellitus is, therefore, important. The sample of the present study consisted of 400 subjects. They were categorized according to residential area and diabetes into four groups: urban diabetic group, urban non-diabetic control group (from a metropolitan city Delhi), rural non-diabetic diabetic group, and rural control group (from villages of Khanpur Kalan, Sonepat, Haryana). Differences in lipid levels and risk factors of emerging cardiovascular diseases between groups were evaluated with analysis of variance. Diabetic patients of both urban and rural areas had significantly higher total cholesterol (TC), triglycerides (TG), very low-density lipoproteins (VLDL), TC to high-density lipoprotein cholesterol (TC/HDL) ratio, TG to high-density lipoprotein cholesterol (TG/HDL) ratio, and atherogenic index (AI) compared to respective controls (p<0.05). The HDL concentrations in urban diabetics were significantly lower (p<0.05) than in urban non-diabetic group and rural diabetic group. Comparison between urban and rural diabetic groups showed significantly higher atherogenic dyslipidaemia (AD) in the urban patient-group (p<0.05). We evaluated significant relationships of diabetes and urbanization with AD by multiple regression analysis. Receiver operating curve (ROC) analysis showed high area under curve (AUC) for TG/HDL in urban diabetic group (0.776, p<0.0001) and in rural diabetic group (0.692, p<0.0001). It is concluded that diabetes was associated with higher AD parameters. Urbanization in diabetes is also associated with elevated levels of AD, indicating higher risk in urban population. This study suggests that TG/HDL may be particularly useful as atherogenic risk predictor in newly-diagnosed type 2 diabetic patients.

## INTRODUCTION

The rising prevalence of diabetes in India (1,2) and other developing countries (3) is chiefly attributed to urbanization. Currently, India is facing a three-fold rise in the prevalence of diabetes in urban as well as in rural areas (4). Among the people of India, the prevalence differs between urban and rural areas (5). The real impact of diabetes is through cardiovascular diseases (CVDs), and diabetic patients are at high risk of developing micro- and macro-vascular complications, with 200% greater CVD risk than non-diabetic individuals (6-8).

Atherogenic dyslipidaemia (AD), characterized by the elevation of plasma total cholesterol (TC), triglycerides (TG), TG-rich very low-density lipoproteins (VLDL), increased low-density lipoprotein cholesterol (LDL), and reduced high-density lipoproteins (HDL) contributes significantly to the excess risk of CVD (9). The newly-addressed lipid profiles: non-HDL, TC/HDL-C, TG/HDL-C, and LDL-C/HDL-C ratios are proposed to be more useful than the traditional ones in CVD risk prediction (10). The Adult Treatment Panel (ATP-III) of the National Cholesterol Education Program has recommended these markers as better predictors of CVD risk in diabetes (11). Some studies suggested lipid abnormalities and higher levels of cardiometabolic risk factors in urban populations compared to rural counterparts (12).

With an ever-increasing incidence of both type 2 diabetes mellitus (T2DM) and CVD in most urban populations, there has been a stressful need for studies that could evaluate risk of cardiovascular disease—the largest cause of death in developing countries (13). Moreover, morbidity and mortality due to CVD at premature age is reported to be high in diabetes (14). Despite high prevalence (4,5,15), there is paucity of studies showing differences in CVD risk factors between urban and rural diabetic population. Moreover, data on AD among diabetic patients in urban and rural areas of developing countries, like India, are uncertain. In addition, there are no definitive reports on the variation of emerging cardiovascular indices, such as TG/HDL and atherogenic index (AI) among urban and rural diabetic patients versus respective non-diabetic controls from north India. Therefore, we studied AD among diabetic patients from urban national capital region versus rural area in north India.

This study was done in two locations: a metropolitan city (New Delhi) and rural villages (District Sonepat) of Haryana, northern India. The primary aims were to see whether the degree of dyslipidaemia and associated rise in lipid risk factors of cardiovascular diseases differed significantly between urban and rural populations newly-diagnosed with T2DM. The changes in diabetes patients were also studied in comparison with controls. The effect of confounders: smoking, alcohol consumption, obesity, hypertension, and family history of CVD on lipid risk factors in diabetes was ruled out by including newly-diagnosed T2DM patients who were non-smokers, non-alcoholics, and normotensives with normal BMI, with the aim of examining whether the AD increases in urban patients versus rural patients of diabetes.

## MATERIALS AND METHODS

### Study subjects and data

This retrospective study was set up to examine the difference in lipid risk factors of cardiovascular diseases among urban and rural individuals with a new diagnosis of T2DM between 1 April 2012 and 30 September 2012. Briefly, the study was nested in the national capital region of Delhi (urban) and the Khanpur Kalan region of Sonepat, Haryana (rural) at BPS Government Medical College. The following information was extracted from reviewing medical records: age, gender, smoking status, alcohol consumption, previous cardiovascular events, BMI, blood pressure recordings, and laboratory results (lipid profile).

All individuals (n=400) were stratified into four groups (100 each) according to diabetes and residential status: (i) urban controls (age 50.3±10.4 years, 50 male/50 female); (ii) urban diabetics (age 52.9±9.2 years, 61 male/39 female); (iii) rural controls (age 49.3±11.0 years, 51 male/49 female); and (iv) rural diabetics (age 51.5±10.0 years, 47 male/53 female). Diabetic patients had fasting blood sugar (FBS) >126 mg/dL according to diagnostic criteria of the American Diabetes Association. The potential confounding variables included extreme BMI, obesity, hypertension, smoking, alcohol-use, menopausal women, parental history of CVD, and hypoglycaemic medication. Because these confounders may influence the results, we excluded them in this study. Patients on antioxidant supplements and hypolipidaemic agents, end-stage renal disease, active infection, pregnant females, chronic or acute illnesses, and patients suffering from endocrine disorders other than T2DM were also excluded. For the purpose of this study, medical records/laboratory entries of all the participants were retrospectively reviewed to obtain relevant data from all subjects.

Plasma levels of fasting glucose, total cholesterol (TC), triacylglycerol (TG), and high-density lipoprotein (HDL) were determined by enzymatic colorimetric methods, using commercial kits. Since the TG level in all participants was lower than 400 mg/dL, VLDL was calculated as TG/5 and LDL, using Friedwald formula (16). Various lipid risk factors of atherosclerosis, such as non-HDL (TC-HDL), ratios of TG/HDL, TC/HDL, LDL/HDL, and non-HDL/HDL, were determined from the lipid profile. AI was calculated as the logarithm of the ratio of concentrations of TG and HDL-C [AI=log (TG/HDL-C)] (17).

### Statistical analysis

Data obtained were presented as mean±SD. Between-group differences for all variables studied were tested by one-way analysis of variance (ANOVA) test. Prior to ANOVA, Levene's test for equality of variances was performed. Parameters with different variances in different groups yielded a positive Levene's test (p<0.05) where logarithmic transformation to the data was applied. The independent associations (if any) of urbanization, diabetes, age, and sex with lipid risk factors of cardiovascular diseases were studied by multiple regression analysis. Receiver operating curve (ROC) analysis was done to predict the atherogenic variables statistically, with higher area-under-curve (AUC) values. A statistical significance was reported at a two-tailed p value of <0.05. All the statistical analyses were done using the Excel spread sheets for Microsoft and MedCalc software (version 12.1.0) (MedCalc®, Mariakerke, Belgium).

## RESULTS

Compared to controls, there was a significant change in FBS, lipids, and atherogenic lipid risk factors among urban and rural diabetic patients. There was also a significant change between urban and rural diabetic groups with the difference being more in the former group (Table 1). Lipids, such as TC, TG, and VLDL, were significantly (p<0.05) increased with a non-significant rise in LDL in both categories of diabetic patients compared to their respective control population. All these variables were not significantly different between two diabetic groups. The HDL level was found to be decreased in both diabetic groups versus their respective controls. The decrease was statistically significant in urban patient-group when compared with urban control group and rural diabetic group (p<0.05). All lipid risk factors were significantly elevated in both urban and rural patients versus their respective controls (p<0.05). All these variables presented higher values in the urban diabetic patients when compared with their rural counterparts (p<0.05).

To study the factors affecting the observed results, multiple linear regression analysis was performed with lipids and lipid risk factors as dependent variables and age, sex, diabetes, and urbanization as independent variables. There was no significant association of age and sex with the results observed. It was found that atherogenic dyslipidaemia and higher values of risk factors were associated with urbanization and diabetes (Table 2).

**Figure 1.**
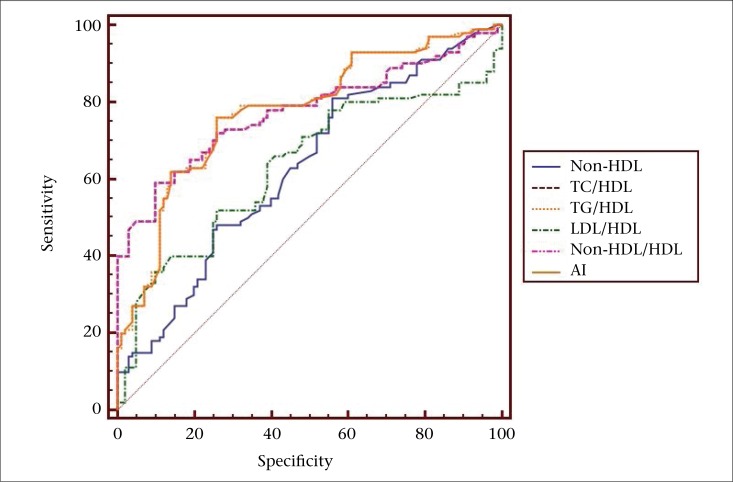
Comparison of receiver operating curve analysis of atherogenic lipid risk factors, showing high area-under-curve value for TG/HDL-C (AUC 0.776, p<0.0001, criterion value >4.1) in urban diabetic group

**Figure 2.**
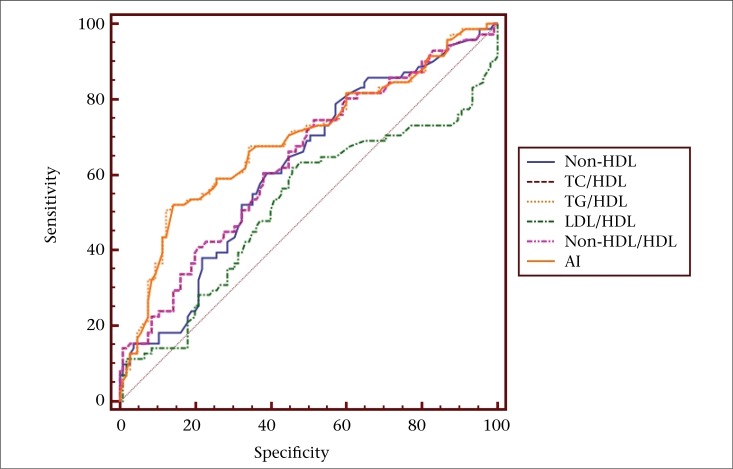
Comparison of receiver operating curve analysis of atherogenic lipid risk factors, showing high area-under-curve value for TG/HDL-C (AUC 0.692, p <0.0001, criterion value >5.0) in rural diabetic group

The ROC analysis yielded AUC values to identify optimum cutoff for predicting CVD risk in both urban and rural diabetic patients. The results obtained were depicted in Table 3. There were statistically significant AUC values for lipid risk factors in urban and rural patient-groups. Comparison of ROC analysis showed that, among all lipid risk factors, the TG/HDL ratio had high AUC values in urban and rural populations as shown in Figure 1 and 2 respectively.

## DISCUSSION

Our results are in agreement with the recent study by one of us (VS Reddy) reporting AD among the newly-diagnosed T2DM patients compared to controls (17). The present study is novel in its approach of addressing the differences in AD between diabetic patients of urban and rural residences of northern India.

**Table 1.** Biochemical characteristics of the study population

Variable	Urban controls	Urban diabetics	Rural controls	Rural diabetics
FBS (mg/dL)	88.94±10.50	148.39±38.22*	91.05±11.77	162.80±57.24¶§
TC (mg/dL)	202.54±43.30	219.05±51.56*	199.74±44.19	222.46±56.62¶
TG (mg/dL)	157.63±66.50	218.65±85.38*	157.33±67.62	215.42±84.48¶
HDL (mg/dL)	43.41±6.34	38.15±8.35*	44.06±6.65	45.30±6.70§
LDL (mg/dL)	126.09±44.24	132.64±52.99	126.31±44.59	131.21±50.12
VLDL (mg/dL)	31.42±13.32	43.81±17.11*	31.45±13.56	43.14±16.92¶
Non-HDL	159.13±38.32	180.90±48.63*	155.69±38.94	177.17±51.84¶
TC/HDL	4.65±0.61	5.90±1.48*	4.51±0.60	4.89±0.80¶§
TG/HDL	3.67±1.56	6.02±2.83*	3.59±1.48	4.83±1.90¶§
LDL/HDL	3.00±0.80	3.36±1.04*	2.83±0.69	2.87±0.86§
Non-HDL/HDL	3.65±0.61	4.90±1.48*	3.51±0.60	3.89±0.80¶§
AI	0.53±0.18	0.73±0.20*	0.52±0.17	0.65±0.19¶§

*p<0.05 between urban controls and urban diabetic groups;

¶p<0.05 between rural controls and rural diabetic groups;

§p<0.05 between urban and rural diabetic groups; AI=Atherogenic index; FBS=Fasting blood sugar; HDL=High-density lipoprotein; LDL=Low-density lipoprotein; Non-HDL=TC-HDL; TC=Total cholesterol; TG=Triglycerides; VLDL=Very low-density lipoprotein

**Table 2.** Results of multiple regression analysis in the study population

Dependent variable	Independent variable					
	Diabetes			Urbanization		
β	SE of β	p	β	SE of β	p
TC	18.62	5.10	0.0003	-1.54	5.28	0.76
TG	60.50	7.96	<0.0001	2.68	8.24	0.74
HDL	-2.32	0.76	0.0025	-3.66	0.79	<0.0001
LDL	-2.60	5.02	0.60	-1.13	5.20	0.82
VLDL	12.24	1.59	<0.0001	0.49	1.65	0.76
Non-HDL	20.94	4.64	<0.0001	2.11	4.80	0.65
TC/HDL	0.84	0.10	<0.001	0.51	0.10	<0.0001
TG/HDL	1.86	0.21	<0.0001	0.62	0.22	0.005
LDL/HDL	0.18	0.09	0.038	0.27	0.093	0.0031
Non-HDL/HDL	0.84	0.10	<0.0001	0.51	0.10	<0.0001
AI	0.17	0.01	<0.0001	0.05	0.02	0.017

AI=Atherogenic index; HDL=High-density lipoprotein; LDL=Low-density lipoprotein; Non-HDL=TC-HDL; SE=Standard error of coefficient; TC=Total cholesterol; TG=Triglycerides; VLDL=Very low-density lipoprotein; β=Coefficient; p=Statistical significance

**Table 3.** Receiver operating characteristic curves for atherogenic lipid risk factors in urban and rural population

Urban controls vs Urban diabetics				Variable	Rural controls vs Rural diabetics			
ROC characteristics					ROC characteristics			
p value	AUC	Sensitivity/ Specificity	Criterion		Criterion	Sensitivity/ Specificity	AUC	p value
0.0012	0.628	81/44	>145	Non-HDL	>141	79/43	0.622	0.0044
<0.0001	0.775	59/90	>5.4	TC/HDL	>4.4	74/48	0.637	0.0014
<0.0001	0.776	76/74	>4.1	TG/HDL	>5.0	51/87	0.692	<0.0001
0.0011	0.632	52/74	>3.2	LDL/HDL	>2.7	62/54	0.520	0.671
<0.0001	0.775	59/90	>4.4	Non-HDL/HDL	>3.4	74/48	0.637	0.0014
<0.0001	0.775	76/74	>0.61	AI	>0.60	52/85	0.691	<0.0001

AI=Atherogenic index; AUC=Area under curve; HDL=High-density lipoprotein; LDL=Low-density lipoprotein; Non-HDL=TC-HDL; TC=Total cholesterol; TG=Triglycerides; VLDL=Very low-density lipoprotein; p=Statistical significance

Dyslipidaemia is one of the major risk factors of CVD in diabetes (18). We observed significantly higher levels of lipids (TC, TG, and VLDL) and all lipid risk factors in both urban and rural diabetic patients compared to their respective controls. In this study, the LDL levels increased, though not significantly, in both urban and rural diabetic patients. Although LDL might not be higher, its metabolism is abnormal in T2DM (19-21). Further, T2DM increased the risk of CVD mortality independent of LDL levels, adding to the greater overall cardiovascular risk in this population (22). Furthermore, studies among northern Indians have shown high TC, TG, and low HDL as the most common lipid abnormalities, with high prevalence of low HDL (12,23-24). There was a decrease in HDL level in both the patient-groups but the decrease was significant only in urban diabetics group compared to the urban control group. These findings clearly indicate atherogenic changes among diabetic patients.

It has been proposed that the emerging markers of CVD risk, such as non-HDL, ratios of individual lipids to HDL, and AI could serve as sensitive markers of insulin resistance, surrogates of small dense LDL, beta-cell function and are better independent predictors of atherosclerosis than the individual lipid parameters (25-29). AI has been proposed as a simple means to estimate AD and the residual cardiovascular risk in T2DM even when LDL is at or below targets in T2DM (27). In accordance with the above observations, the significantly increased lipid risk factors in our study indicate the presence of atherogenic risk in newly-diagnosed T2DM patients. In addition, we propose different criteria for lipid risk factors for predicting CVD risk in urban and rural diabetic patients (Table 3). Among all lipid risk factors, high AUC values were observed for TG/HDL, indicating the importance of this ratio as a cardiovascular risk predictor even in the early stages of T2DM. We found that the TG/HDL ratio showed high AUC values of 0.776 (p<0.0001) in urban diabetics group (Figure 1) and 0.692 (p<0.0001) in rural diabetics group (Figure 2), with a criterion values of >4.1 and >5.0 respectively.

Furthermore, atherogenic risk factors were significantly high in urban patients versus rural patients, suggesting increased risk with urbanization. Recently, Ramachandran *et al.* (1), reported high cardiovascular risk factors associated with urbanization in India. It is clear from our data that these differences in lipid risk factors between urban and rural patient-groups might be attributable to significantly decreased HDL level in urban diabetics group versus rural diabetics group. Therefore, particularly higher levels of AD in the urban group are clinically meaningful owing to the changes in HDL. The most common abnormality found in diabetes is high TG with low HDL—the hallmark of AD (27). Low HDL levels are often accompanied with elevated TG levels as seen in this study and others, and this combination has been strongly associated with an increase in risk (30,31). Recent evidence suggests that increased VLDL in diabetes results in high levels of atherogenic remnants and lower levels of athero-protective HDL, causing vascular complications (32). Hyperglycaemia increases the risk of microvascular complications while dyslipidaemia, a modifiable CVD risk factor that remains largely uncontrolled in T2DM, is a major risk factor of macrovascular complications (33,34). In addition, hyperglycaemia progressively increases the transfer of cholesterol esters from HDL to VLDL, diminishing HDL levels (35). Increased HDL catabolism, free fatty acid flux, and impaired lipoprotein lipase results in higher TG levels, hypercholesterolaemia and lower HDL levels (18,30,36).

It has been reported that T2DM is an independent risk factor of CVD, and the risk is three- to four-fold high compared to non-diabetic population (37,38). To better study the effect of urbanization on AD, we performed the multiple regression analysis showing independent association of diabetes with TC, TG, HDL, VLDL and non-HDL levels. The higher levels of TC/HDL, TG/HDL, LDL/HDL, non-HDL/HDL, AI, and lower levels of HDL in our study population were associated significantly and independently with both diabetes and urbanization. This clearly suggests that, in addition to diabetes-associated increased atherogenic lipid risk factors, urbanization showed independent effect over increased lipid risk factors in diabetes. We could not observe any significant difference in age and sex between diabetic and non-diabetic groups nor could these variables be associated significantly with lipid abnormalities in a multiple regression analysis.

Urbanization leads to unhealthy changes in lifestyle, thus adversely affecting metabolic changes leading to a two-fold increase in diabetes risk in urbanized areas of India than rural India due to industrial development and lifestyle changes (4,39,40). In this study, we excluded smokers, alcoholics, and abnormal BMI to nullify their effect on the results. However, our study has certain limitations, such as lack of data on insulin resistance. Nevertheless, as has been well-documented previously, increased TG/HDL-C and AI, which we have also observed in the present study, may serve as sensitive markers for insulin resistance.

### Strengths and limitations

We do not have data through direct interviews or questionnaire on diet, physical exercise, education, occupation, and sedentary activity, this being the limitation of the study. As we investigated differences in lipid risk factors in a retrospective study, we cannot rule out the fact that physical activity might influence results. Recent studies have reported that exercise and habitual physical activity effectively improves lipid abnormalities, increasing HDL levels in diabetes (41,42). Most of them in the rural group were farmers requiring a lot of physical activity. On the other hand, most of the participants in urban group might have been engaged in work that would require less physical activity compared to rural participants. This may have probably accounted for the higher levels of lipid risk factors in the urban diabetic population compared to that of their rural counterparts.

However, the rural region in this study is a developing region that lies close to urban region called national capital region of India. Recent evidence suggests that developing rural parts of the country are following the transition of urban India with high rates of diabetes and associated high mortality attributable to cardiovascular risk (43). In light of this, our study reporting the differences of various lipid risk factors among urban and rural population is, therefore, important in view of urban transition which may occur in the rural region of this study, which is 100 km close to urban national capital region of India. It is noteworthy that our study is of much importance in line with the recent reports from north India, showing urban-rural differences in lipid profile and the urban way of living, leading to an increase in the prevalence of the well-known risk factors of coronary heart disease (44,45).

### Conclusions

AD was found in diabetic patients of both urban and rural residence, with higher levels of lipid risk factors in the urban patients. The rise in lipid risk factors was associated independently with diabetes and urbanization in our study population, indicating increased risk with urbanization. This would suggest that these patients certainly require physical exercise, diet and lifestyle management in addition to therapeutic intervention to correct abnormal values of atherogenic lipid risk factors, importantly targeting hypertriglyceridaemia and/or hypo-HDL-cholesterolaemia. Our study, owing to the independent association of diabetes and urbanization with AD, would direct us and others for further large prospective studies in determining the contribution of diet, exercise, education, occupation, physical activity, and lifestyle patterns to the increased atherogenic lipid risk factors with urbanization.

## ACKNOWLEDGEMENTS

The authors wish to acknowledge the support received from Prof. R.C. Siwach, Director, and Ms Sonia, Lab Technician, BPS GMC, Haryana and also specially acknowledge Dr. Aparna R. Bitla, Associate Professor of Biochemistry, SVIMS, Tirupati, A.P, for valuable advice on statistical analysis.
